# Femtosecond Laser Filamentation for Atmospheric Sensing

**DOI:** 10.3390/s110100032

**Published:** 2010-12-23

**Authors:** Huai Liang Xu, See Leang Chin

**Affiliations:** 1 Key Laboratory on Integrated Optoelectronics, College of Electronic Science and Engineering, Jilin University, 2699 Qianjin Street, Changchun, 130012, China; 2 Department of Physics, Engineering Physics and Optics & Center for Optics, Photonics and Laser (COPL), Université Laval, Québec City, QC, G1V0A6, Canada; E-Mail: See.Leang.Chin@copl.ulaval.ca

**Keywords:** femtosecond laser, filamentation, remote sensing

## Abstract

Powerful femtosecond laser pulses propagating in transparent materials result in the formation of self-guided structures called filaments. Such filamentation in air can be controlled to occur at a distance as far as a few kilometers, making it ideally suited for remote sensing of pollutants in the atmosphere. On the one hand, the high intensity inside the filaments can induce the fragmentation of all matters in the path of filaments, resulting in the emission of characteristic fluorescence spectra (fingerprints) from the excited fragments, which can be used for the identification of various substances including chemical and biological species. On the other hand, along with the femtosecond laser filamentation, white-light supercontinuum emission in the infrared to UV range is generated, which can be used as an ideal light source for absorption Lidar. In this paper, we present an overview of recent progress concerning remote sensing of the atmosphere using femtosecond laser filamentation.

## 1. Introduction

Laser-based spectroscopic techniques, such as differential absorption light detection and ranging (DIAL), tunable diode laser absorption spectroscopy and laser-induced fluorescence, have been extensively employed for sensing atmospheric trace species because of their high sensitivity, non-intrusiveness and real-time analysis [1–3]. However, conventional laser remote sensing techniques usually require coherent sources with a particular wavelength depending on the species to be sensed, that is, the laser may only be optimized for one pollutant at a time. For example, in the DIAL technique (see, e.g., [1]), the laser light has to be alternatively switched to an on-resonance wavelength, where the species under investigation absorbs, and to a neighboring off-resonance wavelength, where the species does not absorb. Although this technique has been extensively used to measure the pollutants like NO, SO_2_, O_3_, Hg, CH_4_, and benzene with sensitivities in the ppb-range, the laser wavelengths required are over wide spectral ranges, which are not achievable with only one laser source. In particular, for biological agent detection, the UV laser-induced fluorescence Lidar technique is usually used, where broadband spectra are generated. However, such broad band fluorescence represents a weakness insofar as efficient and effective identification of bio-molecules is concerned.

On the other hand, nanosecond laser-induced breakdown spectroscopy (LIBS) is a powerful technique for rapid, on-line and multi-elemental material analysis with only one laser source (see e.g., [4]). It has also been suggested as a potential technique for identifying biological agents [5,6]. This technique is based on the emission spectroscopy of materials ablated into a small plasma by a tightly focused laser beam, generally a Q-switched Nd:YAG laser. However, a key aspect of remote LIBS is to ensure that sufficiently high laser intensities are achieved at a remote position to induce a plasma breakdown on the sample. Clearly, because of diffraction, the proportionality of the focal diameter to the distance leads to the difficulty in delivering high laser intensities over long distances, which limits the operation range of conventional nano-LIBS.

Recent advances in high-power femtosecond laser technologies have made it possible to use only one laser source for simultaneously sensing of multi-pollutants. High peak powers of femtosecond laser pulses with pulse widths of less than 50 fs are commercially available and can be made to be relatively compact. When powerful femtosecond laser pulses propagate in air, filaments appear as a result of the dynamic balance between Kerr self-focusing and defocusing by both the plasma produced by multiphoton/tunnel ionization of the air molecules [7–12]. The equilibrium limits the laser intensity inside the filament core to about 5 × 10^13^ W/cm^2^ (intensity clamping) [13–15]. This peak intensity inside the filament is high enough to dissociate/ionize other gas molecules [16,17], generate higher harmonics [18–21], induce other parametric processes [22] as well as THz radiation [23–27], and explode dust particles and aerosols or induce partial breakdown on solid targets [28,29]. In addition, the non-linear propagation of powerful femtosecond pulses can also lead to the white-light emission with an extraordinary broad spectral content ranging from IR to UV [30–37]. In particular, it is worth stressing that filamentation can even be formed at a far distance in an adverse atmospheric environment [38], and hardly disturbed by turbulence [39–41]. Moreover, the filamentation of powerful femtosecond pulses can be controlled to occur at a distance as far as a few kilometers in the atmosphere [42]. Such properties of the filaments make them a promising technique with high potential in view of applications in remote sensing of trace chemical and biological agents and pollutants in the atmosphere [10,43].

In this paper, we will mainly present two filamentation-based schemes proposed for remote sensing of multi-pollutants. One of the schemes is to utilize the ultra-broad spectral content of the self-transformed white-light laser pulses during laser filamentation [7,44]. In air this white light laser, popularly called supercontinuum, provides an ideal light source for absorption Lidar [45]. The second one is to utilize the high intensity inside the filament (5 × 10^13^ W/cm^2^). Such peak intensity in the filament is high enough to dissociate/ionize gas molecules, to explode fine particles (dusts and aerosols), and to induce a so-called breakdown on solid targets, resulting in the emission of characteristic fluorescence spectra (fingerprints) from the excited fragments, atom or ions. The fluorescence emissions are ‘clean’ and practically free of plasma continuum. It is a universal strong-field phenomenon inside the filament for all targets ranging from gases, aerosols to solids [17,46,47], and thus can be used for identifying various substances including chemical and biological species in the atmosphere [48].

The present article is organized as follows. In Section 2, we will briefly present the physics responsible for the formation and termination of filamentation in air. This is followed in Section 3 by an overview of recent progress concerning remote sensing of atmosphere using filament-induced fluorescence technique. In Section 4, the analysis of multi-parameter atmosphere by using filamentation induced white-light continuum is briefly reviewed. Finally, the summary and conclusions are presented in Section 5.

## 2. Formation and Termination of Femtosecond Laser Filamentation in Air

The basic physical mechanisms of femtosecond laser filamentation in transparent media are now basically understood (see e.g., the review papers [7–12] and references therein). The propagation of femtosecond laser pulses in air is dominated by the dynamical balance between the Kerr effect which focuses the beam and creates a plasma and the de-focusing of laser pulses by the plasma. This equilibrium leads to the formation of self-guided stable channels of plasma with diameters of 100–200 μm called filaments. It was shown that self-focusing occurs only when the input laser power, *P*_in_, exceeds a critical threshold (critical power), *P*_c_, where *P*_c_ = 3.72λ^2^/8π*n*_0_*n*_2_ with *n*_0_ and *n*_2_ the linear index of refraction and second order nonlinear index coefficient, respectively [49]. In air, the critical power for self-focusing was recently measured to be about 10 GW for a 42 fs laser pulse, and it gradually decreases to about 5 GW for a chirped pulse with duration longer than 200 fs [50]. However, it was also found that in some cases the filamentation would be terminated even if the laser power is higher than the critical power due to the diffraction of laser pulses by the plasma produced in the filamentation process, which indicates that the remaining self-focusing power could not overcome the divergence of the laser beam [51].

Furthermore, it was demonstrated that the low intensity background energy (energy reservoir) surrounding filaments plays an important role in their formation [52–55]. When particles like water droplets, snow crystals and dusts with dimensions comparable to or larger than the filament diameters block the filament in the propagation path of laser pulses, the energy in the reservoir will refill the hot core (replenishment) [52,56–58]. Such filamentation properties clarify why filaments can be formed and propagate under adverse atmospheric conditions such as rain, compared to the linear propagation of the beam. The effect of energy reservoir on the formation of filaments was also observed by examining whether the filament would be terminated or not after blocking the background energy [59,60]. It was found that the background can contain up to 90% of the pulse energy, which is benefitial for maintaining the filament formation [61], and thus the filamentation process could be terminated immediately after the energy reservoir was blocked [62]. As a consequence, for the filament formation it is more critical to avoid a diffraction of energy at the edges of the background than a collision with a (small) droplet near the center.

## 3. Femtosecond Filament-Induced Fluorescence Technique

### 3.1. Clean Fluorescence

**Figure 1.**
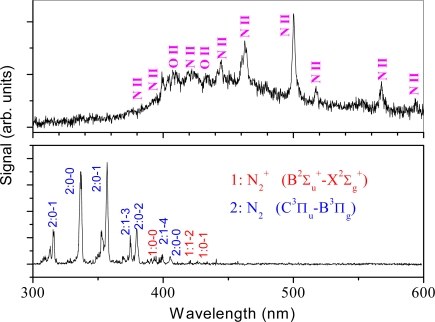
Long pulse-induced breakdown and short pulse filament-induced fluorescence spectra of air obtained in the ambient atmosphere. For both cases, the energy per pulse was 5 mJ. The durations of the short and long laser pulses were 42 fs and 200 ps, respectively. The short and long laser pulses were focused into air respectively by lens with a focal length of f = 100 cm and f = 5 cm. The lines superposed on the continuum shown in the upper spectrum mainly come from the emissions of singly charged nitrogen and oxygen atoms, while the spectral bands shown in the bottom spectrum result from neutral and ionic molecular nitrogen.

The fluorescence emission from the plasma-filament was first shown for nitrogen molecules in air, and it was found that the fluorescence spectra were very clear and free of plasma continuum [16], as shown in Figure 1, in which a 200 ps long (upper) and 42 fs short (bottom) laser pulse was focused in air.

**Figure 2.**
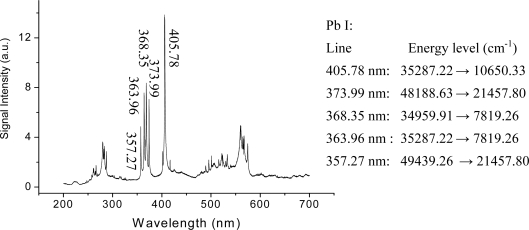
Filament-induced lead spectrum recorded with a Lidar configuration. The sample was located at 2.8 m away from the focal lens, and the gate width of the ICCD detector was 2 μs and the delay time was −3 ns with regard to the laser arriving time on the lead target. As an example, several strong lines in the spectrum are assigned to Pb I.

The clean filament-induced fluorescence spectrum in air may originate from the fact that free electrons induced by the short laser pulse in the plasma filament do not have enough time to absorb more energy from laser photons through inverse Bremsstrahlung [63], and that the plasma density (∼10^14^–10^16^ cm^−3^) and temperature (∼5,800 K) are very low in the filament plasma [64,65]. This also leads to a simple recombination picture of electrons and ions in the plasma filament for the emission of the second positive band system (C^3^Π_u_–B^3^Π_g_ transition) of N_2_, which was ascribed to the primary reaction N_2_^+^+N_2_→N_4_^+^ followed by the recombination with the electron (N_4_^+^ + e→N_2_* + N_2_) resulting in the excitation of the neutral excited state N_2_* (C^3^Π_u_) [65]. While the fluorescence emission from the first negative band system (B^2^Σ_u_^+^ –X^2^Σ_g_^+^ transition) of N_2_^+^ was theoretically ascribed to intense laser-induced multiphoton or tunnel ionization of inner-valence electrons of neutral nitrogen molecules, leaving the molecular ion N_2_^+^ in the excited state B^2^Σ_u_^+^ [66]. Filament-induced clean fluorescence emission from the solid target was also observed, as shown in Figure 2, in which the lead spectrum was recorded in the region of 200–700 nm with the laser pulses focused in air using a fused silica lens (f = 5 m). The lead sample was fixed on a rotating stage to provide new, unprocessed material for successive laser shots, and the sample was placed 2.8 meters away from the focal lens and perpendicular to the laser beam [47]. The clean fluorescence spectrum induced by femtosecond laser pulse filamentation in air was also ascribed to the low temperature and electron density, which was determined respectively to be 8 × 10^17^ cm^−3^ and 6,794 K. Because of the short pulses and the relatively low plasma temperature, the continuum emission associated with the plasma itself, *i.e.*, free-free or free-bound transitions that result from collisions between electrons and ambient gas species and electron-ion recombination in the plasma, is much weaker when compared to conventional ns-LIBS [47].

Thus, filamentation together with clean fluorescence constitutes the key towards identifying various targets (gases, solids or aerosols) with very good resolution using only one laser.

### 3.2. Application to Samples in Gas Phases

#### 3.2.1. Detection and identification of gas molecules

**Figure 3.**
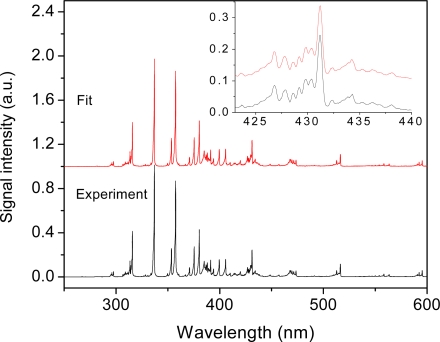
Filament-induced fluorescence spectrum of air containing 1,316 ppm of C_2_H_2_ and 5,263 ppm of CH_4_ with the fit spectrum by a genetic algorithm under the assumption that the trace species and concentrations in the mixture are unknown. The fit spectrum is shifted in order to facilitate the observation. The inset shows part of the experimental and fitting spectra in a higher resolution. The calculated concentrations of acetylene and methane are 1,592 and 6,342 ppm, respectively. The spectra shown in Figure 3 come from the mixture of air, C_2_H_2_ and CH_4_. Some spectral lines such as CH result both from C_2_H_2_ and CH_4_ [67].

The clean fluorescence spectra were later demonstrated from the emission of fragments of fluorine-containing halocarbons, notably HCFC-124, CF_4_ and C_2_F_6_, mixed with air in a cell at atmospheric pressure [17]. Subsequently, simultaneous detection and identification of two unknowns (using methane and acetylene as test targets) mixed with air at atmospheric pressure were performed using filament-induced nonlinear spectroscopy (FINS) [67]. A genetic algorithm was used to identify the unknown spectra with the premise that the spectral database including the spectral signatures and the strengths of the signals of the corresponding trace species was built, as shown in Figure 3. It was found that the detection sensitivity could reach the ppm to ppb level, depending on the induced fluorescence efficiency from the molecules.

The fluorescence emissions from a variety of hydrocarbon molecules were theoretically attributed to the neutral dissociation of parent molecules via superexcited states, *i.e.*, states beyond the ionization limit, created by multiphoton/tunnel excitation [68]. The idea of neutral dissociation of superexcited states as one of the possible mechanisms responsible for the fluorescence emissions was experimentally confirmed by using a pump-probe technique [69]. When an infrared 1338 nm laser was used as the probe, an unambiguous reduction of the CH fluorescence signal dissociated from CH_4_ was observed, which was attributed to the de-excitation of the super-excited states of CH_4_ by the probe laser pulse. The lifetime of the super-excited state of CH_4_ was measured to be about 160 fs.

**Figure 4.**
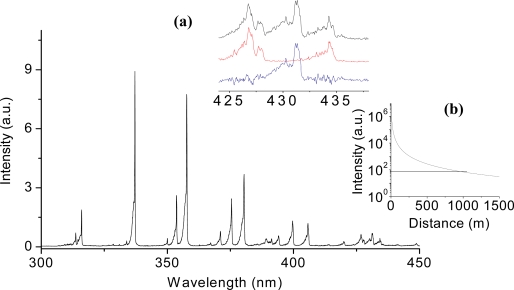
Filament-induced fluorescence spectrum of mixture of CH_4_ and air with a CH_4_ concentration of 2.6% (v./v.). The inset **(a)** shows the spectrum in a higher resolution (top), the spectrum of pure air in atmospheric pressure (middle), and the subtraction of the mixture and pure air spectra (bottom). The band results from the A ^2^Δ-X ^2^Π transition of CH. The inset **(b)** shows the extrapolation of the detection limit according to the LIDAR equation (*I* = *L*/*R*^2^, where *I* is the signal intensity, *L* the effective filament length and *R* the distance between the end of the filament and the detector). The 3σ detection limit was about 0.9 km for the CH_4_ concentration of 5% and the filament length of 20 m, where σ is the standard deviation of the background level.

Remotely, using the FINS technique, the fluorescence emission of nitrogen molecules from the plasma-filament has been extensively studied, not only for the purpose of remote sensing [70,71], but also for characterizing the plasma filament [64,65,72,73]. For gas samples, the FINS technique has so far been applied to CH_4_, C_2_H_2_, C_2_H_4_, ethanol vapor and smoke (from burning mosquito coils in air) [74–78]. For the greenhouse gas, methane [74], well resolved backward fluorescence from dissociated CH radicals measured from a distance of a few meters was used to quantitatively analyze the concentration of CH_4_ as well as its remote detection limit (Figure 4). Based on the experimental results, it was estimated that the concentration sensitivity could be down to the ppm range, and the detection range limit could extend up to the kilometer range. Subsequently, in order to perform such experiments in open-air condition, ethanol was selected as the sample since it is not harmful for human health as compared to most of the hydrocarbon molecules which are toxic and unsuitable. In the ethanol vapor experiment [75], backward fluorescence emission of the ethanol concentration at 0.8% could be clearly observed at a distance of 30 meters using a simple telescope system.

Recently, the remote FINS technique was used to probe a cloud of smoke, which was produced from burning mosquito coils located at a distance of 25 m from the laser source and LIDAR detector [76]. CN, CH and C_2_ molecular fragments were identified in the sample. Most recently, remote sensing of trace methane using FINS was performed in an open field atmosphere during daytime (with strong sun light) [77]. From the comparison of the results of the laboratory and the open field, it seems that the deterioration of the filaments from the atmospheric turbulence, the profile of the laser beam, combined with the varying sunlight background decreased the efficiency to detect the CH fluorescence by a factor of only three to four between the laboratory experiment and the field experiment. In addition, the remote detection of the mixture of CH_4_, C_2_H_2_ and C_2_H_4_ at 118 m distance was performed [78]. The measurements mentioned above were performed with a single near-infrared femtosecond laser, illustrating the possibility to induce characteristic fluorescence from a large number of molecular species. These results open the way to multiple species analysis in atmospheric sensing by combining FINS technique with suitable fluorescence collection systems.

#### 3.2.2. Stabilization and enhancement of fluorescence

However, it was noted in the study of remote nitrogen fluorescence spectra induced by the FINS technique that the nitrogen fluorescence signals fluctuate significantly on a shot-to-shot basis despite the rather stable laser pulse energy with an initial large diameter-collimated terawatt level femtosecond laser beam (∼25 mm) [54]. It was observed that the signal intensity distribution along the propagation path as well as the starting point of the filamentation varies randomly. This was mainly ascribed to the competition among different filaments [79], which originate from inhomogeneous intensity distribution in the transverse cross section of the laser pulse due to either initial laser imperfections arising from the source itself or during the propagation through any non-homogeneous optical medium. These ultimately lead to the formation of multiple filaments that are co-propagating in air [79–83]. Since in atmospheric remote sensing it is of particular importance to increase the signal-to-noise ratio, it is necessary to overcome such fluctuations of fluorescence induced by the femtosecond laser filamentation. We will in the following introduce several examples which have been proposed to stabilize and enhance fluorescence signals by controlling the filamentation processes.

The initial laser beam diameter was found to have an effect on the fluorescence signals from nitrogen molecules in air [54,84]. It was observed that when the initial beam diameter of the collimated femtosecond laser pulse was decreased from 25 mm to 8 mm by keeping the input laser pulse energy constant the measured backscattered fluorescence signal could be enhanced by three orders of magnitude [54]. Numerical simulations showed that in the small beam there is a faster growth of multiple filaments with propagation distance, a larger average diameter of plasma channels, and a larger overall amount of electrons in the transverse beam section [55]. The increase of the signal and its stability associated with beam squeezing is due to the more effective usage of the background energy in the small beam. On the other hand, it was found that by inserting an iris at the beginning of the propagation path of initially unfocused pulse in air, the N_2_ fluorescence signal and the length of femtosecond laser filaments formed can be significantly increased when the diameter of an aperture was adjusted to an optimal value [84]. Theoretical 3D+ time stochastic numerical simulations showed that the optimum aperture size corresponds to the case where multiple filaments concentrate around the propagation axis to interfere and form a regularized elongated structure with higher overall amount of plasma.

The fluorescence signals of N_2_ could also be enhanced by merging the multiple filaments into a geometrical focus [85]. It was found that when a terawatt level laser pulse propagates in air some hot spots in the beam profile are unavoidable to self-focus in air at a short distance. By using a telescope to enlarge the diameter of the initial laser beam, thus enlarging that of the hot spots, such early self-focusing could be overcome. In this manner, the effective focal length of the laser beam can be controlled to be much shorter than the self-focusing distance of both the enlarged beam and the hot spots, leading to the merging of multiple filaments into the geometrical focus. Since in this experimental scheme filamentation starts near the geometrical focus and the beam size is small at this position, the more effective usage of the background energy in the smaller beam results in consistent and strong nitrogen fluorescence signals. The control of femtosecond laser filamentation by a telescope was also performed by another group in [86].

Similarly, the fluorescence emission of N_2_ was found to be significantly enhanced by modifying the initial divergence of the laser pulse using adaptive optics [78,87]. A specially designed focusing telescope including a deformable mirror that corrects the wavefront’s aberrations working in a closed loop system with a wavefront sensor can deliver laser pulses over long distances and generate powerful filaments. The collapse distance increases with the beam divergence. Using this configuration, strong nitrogen signal was generated at a distance as far as 90 meters using 40 mJ laser pulses [87]. This technique was also used for detecting the CH_4_ and C_2_H_2_ samples at a distance of about 110 meters from the focusing beam expander [78].

For the detection and identification of trace gas in air, one should overcome the spectral interference arising from the N_2_ fluorescence. It was found that the characteristic fluorescence of atmospheric pollutants usually has a lifetime longer than that of nitrogen and the pollutant fluorescence could thus be isolated from the background such as the nitrogen fluorescence by temporal gating in the detection [17,67]. This technique is certainly at the expense of a reduction in the fluorescence detection sensitivity but with a substantial gain in signal-to-background ratio. Besides the time-resolved measurements, it is also possible to first record the background reference signals without the pollutants, and then obtain the clean fluorescence spectrum of pollutant molecules by removing the reference signals. In this case, the detection sensitivity will not be influenced, as presented in [67]. Moreover, another major noise source for the detection of pollutants using the filamentation-induced fluorescence technique is the self-transformed white light laser (supercontinuum) as a result of the filamentation process [44]. The white light may mask the fluorescence signals generated by the pollutant being targeted. However, it was experimentally shown that the fluorescence spectrum emitted inside the filament could be distinguished from the backscattered supercontinuum both spectrally and temporally over a long range in atmospheric air [85,88] by generating strong and short filaments at a remote focus [85].

### 3.3. Application to Solid Targets

LIBS (laser-induced breakdown spectroscopy) using femtosecond laser pulses called fs-LIBS has recently attracted much attention because of its promising properties, including lower ablation energy threshold, low continuum emission, higher sensitivity, and improved detection precision. However, most fs-LIBS experiments were performed with tightly focused laser beams only at short distances (see e.g., [89–91]). The high intensity (∼5 × 10^13^ W/cm^2^) in the filament can remotely ablate the solid target into a small plasma, which emits the fluorescence from excited atoms, ions or molecular fragments. Since 2004, remote filament-induced breakdown spectroscopy (R-FIBS), a special configuration of fs-LIBS, has been developed for remote elemental analysis of metallic [28,92] as well as biological samples [93].

#### 3.3.1. Detection and identification of metallic samples

Using R-FIBS, the fluorescence spectra of copper (Cu) and iron (Fe) targets located at a distances of ∼90 meters were first obtained [28], and later the fluorescence emission of the Cu target placed at a distance up to 180 meters was demonstrated [94]. The results showed main features of filament-induced ablation on the surface of metallic samples and associated plasma emission. The R-FIBS technique was also used to detect aluminum and lead samples with a telescope and adaptive mirror as the laser sending systems at a distance of 50 and 118 meters, respectively [78,92]. Besides using the infrared 800 nm laser as the light source, similar experimental demonstration of R-FIBS using UV laser at 248 nm with pulse duration of 450 fs and energy of up to 20 mJ was performed for the detection and identification of brass, lead and different types of stones related to the objects of cultural heritage [95]. These observations revealed the remarkable property of FIBS for the detection and identification of pollutants over a long distance independently of the diffraction limit of laser beam.

#### 3.3.2. Detection and identification of chemical and biological targets

**Figure 5.**
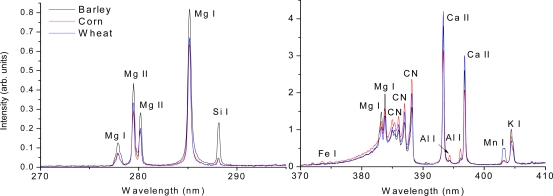
An example of FIBS spectra in the ranges of 270–295 nm and 370–410 nm obtained from the barley, corn and wheat grain dusts with the delay of t = 60 ns with respect to the laser pulse on the target (t = 0). Laser pulse energy was 7 mJ and the ICCD gate width was 2 μs. The Mn, Si, and Al lines resulting from the three samples show a large difference in signal intensities.

The R-LIBS technique has also been applied for the detection of solid chemical-biological samples. The remarkably distinct spectra of egg white and yeast powders located 3.5 m away from the detection system were experimentally shown in [93] by using the FIBS technique. The same fluorescence spectrum of yeast has also been obtained successfully when the sample was located at a distance of 50 meters from the laser source as well as the detection system. In particular, by using the FIBS technique, the feasibility of remote detection and differentiation of some very similar agriculture related bioaerosols, namely barley, corn, and wheat grain dusts was also demonstrated [29]. All the species showed identical spectra, namely those from molecular C_2_ and CN bands, as well as atomic Si, C, Mg, Al, Na, Ca, Mn, Fe, Sr and K lines. These identical spectral bands and lines reveal similar chemical compositions; however, the relative intensities of the spectra are different showing different element abundances from these three bio-targets. The intensity ratios of different elemental lines were used to distinguish these three samples, as shown in Figure 5.

Using R-FIBS, dinitrotoluene (DNT) and ammonium perchlorate samples were detected [96], in which the filament-induced fluorescence signals of these materials were produced both by IR (100 fs) and UV lasers (200 ps). Comparison demonstrated that the UV filaments appear to have marked advantages in being able to capture clear spectroscopic signatures of these two materials, in addition to propagating over longer distances. R-FIBS was also used to measure the carbon/clay ratios between three graphite composites of different hardness at a standoff distance of 6 meters [97]. This measurement was performed using both R-FIBS and femtosecond laser induced breakdown (fs-LIBS), and it was found that these two methods revealed similar selectivity and ability to excite emission, but the R-FIBS technique produced more accurate results than those produced by fs-LIBS (tightly focused beam). This was ascribed to the intensity clamping nature of the filament produced in the R-FIBS technique.

#### 3.3.3. Stabilization and enhancement of fluorescence from solid targets

For solid target detection, it was shown that the continuum emission in the fluorescence spectra associated mainly with the white light due to the filamentation in ambient air can be significantly reduced by moving the starting point of the filament with respect to the target surface [47]. This was because the spectral broadening of the supercontinuum develops progressively along the self-induced plasma column in air and the broad backscattered supercontinuum only becomes fully developed at the end of a long plasma column, which is the result of the distance-cumulative effects of self-phase modulation and self-steepening of the fundamental laser pulse [31]. On the other hand, using time-resolved measurement, the filamentation-induced white light noise can be easily minimized [93].

**Figure 6.**
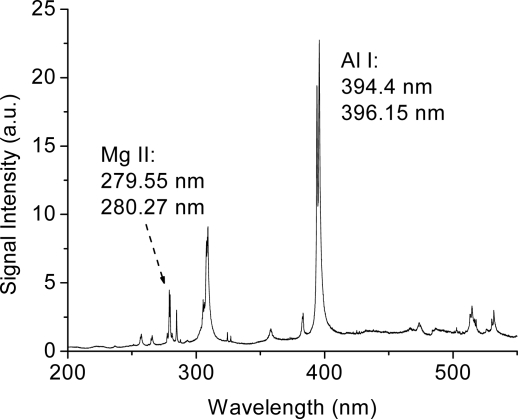
R-FIBS spectrum taken for aluminum sample located 50 m away. The gate width of the ICCD detector was set to 20 μs and the gate was opened 33 ns before the laser pulse arrived at the target.

It was also possible to obtain clean fluorescence by using a telescope as sending optics system. It was shown that the telescope adjusted by varying the relative distance between the divergent and convergent optical components could greatly improve the performance of remote FIBS (R-FIBS) of aluminum targets [92]. The filaments generated were short, resulting in a negligible white light continuum, and finally, realizing a non-gated R-FIBS measurement (Figure 6). In addition, using a specially designed focusing telescope with a deformable mirror included [78], the laser pulse could be properly deliver the laser pulses over long distances and generate powerful filaments by correcting the wavefront’s aberrations working in a closed loop system with a wavefront sensor by the deformable mirror. Using this configuration, strong lead signal was generated at a distance as far as 118 m using 80 mJ/5 ps negatively chirped pulses [78].

### 3.4. Application to Water Aerosols Containing Metallic Salts

Femtosecond laser filamentation in air was also used for probing a cloud of microdroplets in which table salt was dissolved [46,98,99]. By using R-FIBS with Laser pulses of 70 fs duration and 130 mJ energy, Na fluorescence was observed remotely at a distance of 16 m [98]. It was further demonstrated that the fluorescence emission of the saltwater aerosols located up to 70 m away from the LIDAR mirror could be clearly observed by R-FIBS using negatively chirped 10 ps/72 mJ pulses with the telescope designed in [94] as sending optics [46]. It was found that the R-FIBS technique was sensitive to the solvent as well. Four hydrogen bands from the Balmer series were observed in aqueous microdroplet cloud after H_2_O molecules were exploded inside the filaments. Additionally, a cloud of aqueous aerosols containing a mixture of PbCl_2_, CuCl_2_, FeCl_2_ and NaCl were detected using R-FIBS [99]. It was found that fluorescence from all the metallic ions dissolved could be well observed. Moreover, these spectrally narrow atomic transitions excited in the low density plasma did not show any signal overlap. Using the adaptive optics, the fluorescence signals from the aerosol target located 118 meters away from the laser sending systems were observed [78]. These observations exhibited by the characteristic fluorescence of different trace constituents give a good idea of the great potential of the technique for real application remote sensing of aerosols.

Using femtosecond terawatt laser system, riboflavin-containing biological aerosols at a 45-m distance were also detected [100]. However, in this case, the laser intensity in the interaction zone was kept at a level of 10^11^ W/cm^2^ to induce two-photon excited fluorescence of riboflavin. This intensity was two orders of magnitude lower than the clamped intensity inside the filament. It was also demonstrated that for multiphoton induced processes such as multi-photon excited fluorescence [101] and multi-photon ionization [102], the emitted light from the particles was strongly enhanced in the backward direction. This is obviously very advantageous for Lidar remote sensing, since it compensates to a certain extent the decrease in sensitively. This phenomenon was understood as a manifestation of the time-reversal principle. Interestingly, it was also shown that the backward fluorescence of nitrogen molecules and ions generated from femtosecond laser-induced filaments shows an exponential increase with increasing filament length, indicating amplified spontaneous emission [103]. The existence of the gain in the backscattered fluorescence from the filament is particularly important for remote-sensing applications. It is expected that the fluorescence from other molecules inside the filament will undergo amplification as well. This is because from our recent work, the fluorescence coming from neutral fragments of more complex molecules such as CH_4_, O_2_, *etc*. is the result of neutral dissociation through super-excited states [68,69,104,105]. These fluorescing fragments are initially excited at birth (fragmentation) and hence, the population is inverted which should give rise to ASE along the filament (forward and backward). Another way for aerosol Lidar detection using intense femtosecond lasers is to utilize the second and third harmonic generation [106–108] for remote sizing and electric charge measurements on cloud droplets.

## 4. Femtosecond Filamentation-Based White-Light Techniques

The white-light spectrum induced by femtosecond laser filamentation can span from the ultraviolet (UV) to the infrared (IR) (supercontinuum) (see e.g., [7–12] and reference therein). This broadband white light makes it an ideal source for application in remote sensing of multiple constituents in air by combining white light absorption spectroscopy with Lidar technique [45,109], as well as by the scattering of white light [110].

### 4.1. White-Light Absorption Lidar

With the white-light Lidar technique, the absorption spectra of oxygen and water vapor in the atmosphere were shown by using the supercontinuum generated by a so-called “Teramobile” system [111]. The spectra were recorded in the range of visible to near-infrared 850 nm, and the detection was reached up to the distance of 1.1 km [112]. It was found from a comparison between the experimental results and the tabulated spectroscopic data that an excellent correlation with measurements was made on water vapor whereas observations on the oxygen showed discrepancy. Subsequently, the Lidar signals of the supercontinuum in the near-infrared were obtained [32]. A signal up to 4 km in altitude, in the band 1–1.7 μm, was collected using a 2-m astronomical telescope. A 10-fold enhancement of the infrared signal backscattered from the atmosphere compared with that expected using a previously measured laboratory spectrum was observed, which suggested a more efficient frequency conversion into the infrared under long-distance propagation conditions. It was also shown that the temperature and humidity of atmosphere at the altitude of 4.5 km above the ground level could be determined by analyzing the range-integrated spectra of the backscattered white-light continuum between 680 and 920 nm, which covered the rotational-vibrational band of H_2_O centered at 820 nm, as well as the O_2_ A band around 762 nm, showing the ability of multi-parameter analysis of the cloud microphysics by the white-light differential absorption Lidar [113]. In addition, the distribution of the ozone (O_3_) concentration in the range of 500–1,200 m in the atmosphere was monitored at different time of a day using the teramobile system [114].

The feasibility of the filamentation-induced white light continuum for application to remote sensing of trace gases in air was recently tested in the laboratory. The white light continuum in a broad wavelength range from 300 to more than 2,200 nm generated by the filamentation of femtosecond laser pulses in a Kr gas cell was used to perform direct absorption spectroscopy of CO_2_ at around 2,000 nm in a laboratory 9-meter absorption cell [115]. The absorption spectrum from 1,100 to 2,200 nm was shown. It was estimated from the absorption band at 2005 nm that the measured CO_2_ concentrations could reach to an accuracy of 1–2 ppm from the signals collected for the atmospheric optical length of around 5.5 km through the air.

### 4.2. White Light Scattering

Aerosol and cloud layers in the atmosphere could be detected by scattering the white light generated from the filamentation of femtosecond laser pulses in optical media [116]. It was shown that strong signals from an aerosol layer (from 0.5 to 2 km) and a cumulus cloud layer (from 2.2 to 2.3 km) could be observed on the three channels of 350, 550, and 700 nm with the white light source spanned from 300 to 950 nm generated by the filamentation of femtosecond laser pulses in a 9-meter-long krypton tube [116]. The channel with the longer wavelength of 700 nm showed a higher capacity to distinguish the aerosol layer when compared to the 350 nm channel. Subsequently, utilizing the polarization property of the coherent white light continuum, the depolarization remote sensing of aerosol and cloud layers with the channel of 450 nm was performed and backscattered signals corresponding to 0.6 and 1.0 km in height from running clouds were observed [117]. Moreover, simultaneous three-wavelength depolarization measurements of cloud and aerosol at 450, 550, and 800 nm were tested [110], and used to detect the Asian dust particles [118]. It was also shown that particle size and density of water droplets within the cloud could be extracted from the angular multiple scattering profiles of the white-light continuum generated by the filamentation in air [113].

## 5. Summary

In this article, we have reviewed the representative results of remote sensing of atmosphere by techniques based on femtosecond laser filamentation phenomena. The samples include gases, powders, smoke, water aerosols containing multiple solutes, metallic targets, water droplets within the cloud. Experimental techniques include filament-induced characteristic non-linear fluorescence of gases, filament-induced breakdown spectroscopy, white-light absorption Lidar, and multiple scattering of white light continuum. Based on the experimental evidences, we may conclude that a single laser is sufficient to induce the characteristic fluorescence and to obtain absorption bands for a large number of molecular species, showing the possibility of observing many atmospheric constituents of interest. This allows us to qualify the femtosecond laser filamentation as an ideal tool for the detection and identification of atmospheric constituents. This opens up a new pathway towards remote detection of targets related to safety, security and pollution as well as global environmental monitoring such as greenhouse gases.

The size of ultrafast laser sources is constantly shrinking and thus, relatively compact femtosecond remote sensing systems are possible for practical applications. However, most of the experiments presented using filament-induced fluorescence techniques were performed in a laboratory scale. While the white light-based techniques were carried out in outdoor environment, the samples were limited to water droplets and oxygen. This requires much more effort to confirm the precision and reliability of different filamentation-based techniques and to clarify their practical application scopes. In particular, for filamentation-induced fingerprint fluorescence spectra, in view of the complexity of biological matters and of fragmentary knowledge, there are still many challenges ahead not only for practical applications, but also for understanding the underlying physics and chemistry.
